# Selection of individuals who may benefit from pancreatic cancer surveillance

**DOI:** 10.1002/ueg2.12660

**Published:** 2024-11-01

**Authors:** Marco J. Bruno

**Affiliations:** ^1^ Department of Gastroenterology and Hepatology Erasmus MC – University Medical Center Rotterdam the Netherlands

**Keywords:** pancreatic cancer, surveillance

Pancreatic cancer is one of the deathliest cancers and is projected to be the second cause of cancer death in 2030.^1^ Over the last decades, the overall outcome for patients has hardly changed. This is mainly because the disease is usually detected at a late stage when the cancer has already spread beyond the pancreas. Neoadjuvant treatment followed by surgery may improve survival in selected patients, albeit measured in months, not years.^2^ It is for these facts that primary or secondary prevention strategies have gained attraction to avoid cancer from developing or detect pancreatic neoplasia at an early asymptomatic stage.

A misconception commonly present amongst the public and even physicians, is that all surveillance is good. This is far from the truth and already back in sixties of the last century, Wilson and Jungner defined a set of criteria to appraise the validity of a surveillance initiative.^3^ The downsides of surveillance can be substantial. For one, false positive test outcomes result in unnecessary medical interventions with associated morbidity and mortality which is considerable in the case of a pancreatic resection. This along with other considerations outlined in the Wilson and Jungner criteria including that the program should be cost‐effective, make it of the utmost importance to target surveillance to populations that truly benefit. It is here where the article of Maurer et al. fits in nicely and adds important data to the field.^4^

Maurer et al. report the outcome of their 20 years' experience of the surveillance of familial pancreatic cancer (FPC) families in the university clinic in Marburg, Germany. The diagnostic yield for clinically significant lesions, defined as either pancrearic ductal adenocarcinoma, high‐grade pancreatic‐intraepithelial‐neoplasia (PanIN3) and intraductal‐papillary‐mucinous‐neoplasia (IPMN) with high‐grade dysplasia, was 13.5% (10/74) for variant carriers compared to only 0.8% (2/263) for individuals at risk (IAR) of variant‐negative FPC families (*p* < 0.001).

In 263 variant‐negative IAR, a total of approximately 1352 surveillance visits, 1352 MRI/MRCPs and 568 EUS investigations were performed (recalculated from the numbers provided in the manuscript) which signifies a substantial investment in terms of resources. In the 10 variant‐negative IAR in whom a lesion was suspected and underwent surgery, in only 2 a lesion was found considered to be of clinical importance (PanIN 3 + pTisN0M0 [*n* = 1], PanIN 2 + BD IPMN with high grade dysplasia [*n* = 1]). Eight of 10 (80%) underwent surgery for either a neoplastic lesion that is not considered an indication for resection (PanIN 2 [*n* = 1], mucinous cystic neoplasm with low grade dysplasia [*n* = 1]), or a non‐neoplastic lesion (serous cyst [*n* = 3], focal fibrosis and PanIN 1 [*n* = 2], and no pancreatic lesion identified [*n* = 1]).

With two clinically relevant lesions detected amongst 263 persons over a 20‐year period, the high number of costly imaging investigations, and a false positivity rate of 80% resulting in unwarranted pancreatic resections, the cost/benefit ratio of pancreatic cancer surveillance in variant‐negative IAR clearly does not add up.

Can it be concluded from the current study that surveillance in variant‐negative IAR is ineffective and should be abandoned? Based on the data provided in the current study such conclusion would be premature. What it shows is that with the current strategy including the starting age, surveillance it is not effective. The mean age at first screening for the variant‐negative IAR was around 48 years. With a mean follow up of 64 months (for variant carriers and variant‐negative IAR combined) this implies that the current age of individuals still under surveillance is relatively young. It could therefore well be that for the variant‐negative IAR we are too early in the game. A steep incline of pancreatic cancer incidence in the overall population is observed around age 60 with most patients being affected between age 65 and 75. Possibly, the age at which surveillance should start in variant‐negative IAR is 55, rather than 40. Also, the concept that surveillance in family members should start 10 years earlier than the youngest family member diagnosed with pancreatic cancer is debatable. This refers to the concept of genetic anticipation which is characterised by a reduction in the age of disease onset or an increase in severity of clinical features of a genetic disorder in successive generations and has been suggested in several diseases and cancer syndromes.^5^ One of the major concerns when assessing genetic anticipation is ascertainment bias which introduces a possible younger onset age bias as mutation carriers suffering from early onset cancer are more likely to fulfil the criteria for being genetically tested than mutation carriers with late‐onset cancer.

Lastly, it should be acknowledged that the possibility to identify variant‐negative IAR is declining. In the absence of a known mutation the diagnosis of familial pancreas cancer depends solely on whether pancreatic cancer occurred in 2 or more first‐degree relatives. Families are getting smaller and smaller. For example, the estimated average number of children born per woman (total fertility rate) in Europe, has declined from 2.6966 in 1950 to 1.5 in 2024.^6^ It is therefore becoming more difficult to identify an individual at high risk solely based on the number of FPC cases, simply because there are fewer family members that count to be able to fulfil the criteria. This calls for more in‐depth research to identify susceptibility genes for pancreatic cancer and their associated risk.

In the meantime, we should concentrate on trying to make surveillance work for individuals at the highest risk and improve upon imaging diagnosis (artificial intelligence) and biomarkers for the detection of early neoplasia. If we cannot make it work for the highest risk individuals, it will most certainly fail in those at a lesser risk.

In conclusion, the article of Maurer et al. adds to existing reports about the ineffectiveness of current screening practices in variant‐negative IAR^7^ and calls for a revaluation of its indication and criteria.

## CONFLICT OF INTEREST STATEMENT

The authors have no conflicts of interest to declare.

## Data Availability

Data sharing is not applicable to this article as no new data were created or analysed in this study.
